# Acute stress and gut microbiome: a potential *in vivo* rodent model to study molecular and pathological mechanisms

**DOI:** 10.2144/fsoa-2023-0038

**Published:** 2023-04-06

**Authors:** Ruqaiyyah Siddiqui, Naveed Ahmed Khan

**Affiliations:** 1 College of Arts & Sciences, American University of Sharjah, University City, Sharjah, 26666, UAE; 2 Department of Medical Biology, Faculty of Medicine, Istinye University, Istanbul, 34010, Turkey; 3 College of Medicine, University of Sharjah, University City, Sharjah, 27272, UAE

**Keywords:** epigenetics, *in vivo*, microbiome, rodents

Humans are merely one species among countless others, and we have only recently appeared on the earth [1]. Over millions of years, other species have demonstrated their capacity for adaptation, evolution and survival, indicating that humans can learn from them. Animals like crocodiles and alligators, for instance, have lengthy lifespans of up to 100 years despite being regularly subjected to acute stress, radiation, heavy metals, inadequate food and pollution. They also survived the devastating Cretaceous–Tertiary extinction event [2,3]. These species have shown the ability to overcome harsh and stressful environments that are harmful to *Homo sapiens*. Since the reason for their resistance is unclear, it is speculated that these species have defense mechanisms [4].

In addition to playing a role of the immune system and epigenetics, it is likely that the microbial gut flora is critical in maintaining normal physiology and wellbeing of its host [5,6]. In addition to their role in metabolism and immunomodulation, the gut microbiota exact their effect through epigenetic modifications. Mammals that tend to live in close proximity to humans, typically rodents, are routinely exposed to pollution, overcrowding and environmental stresses. Rodents have been around for over 56 million years and are well adapted to withstand unhygienic/harsh environments that are detrimental to human health. Given the challenges of studying molecular mechanisms of acute stress in humans, it is logical to propose the use of rodents to understand pathophysiology of acute stress. Whole organism mammalian models are appropriate to investigate performance under the physiological and behavioural challenges in response to stressful conditions. Here, we discuss the rodent hindlimb unloading model, as a model to study the underlying molecular mechanisms of acute stress. The experimental rodent (such as C57B/6 mice) model of ‘hind-limbs unloading (HLU)’ recapitulates several features of stress, muscle atrophy and weakness. The age of 4 months equates to a mature adult human of 25–30 years of age [7,8]. In this model, the hindlimbs of rodents, particularly mice and rats, are elevated so that a head-down tilt of approximately 30° occurs, thus causing headward shifts in fluids and unloading of the weight from the hind limbs. A 30° angle of unloading is used as it is sufficient to provide normal weight-bearing on the forelimbs and unloads the lumbar vertebre. The rodent hindlimb unloading model has been used to study cardiovascular, musculoskeletal and metabolic changes as well as for examining the response of other physiological systems to unloading and the recovery from unloading (detailed in [9]). Rodents are maintained in a controlled environment (room temperature of 20–22°C, with light/dark cycles lasting 12 h each) and are given access to food (a typical mouse chow diet) and water as needed. A thin string with one end fastened to the rodent’s tail and the other to the top of the cage is used to suspend each mouse in its own cage for the HLU group of mice [10]. Animals are monitored strictly on a daily basis for body weights, food and water intake, physical activity and other signs of distress as described in detail elsewhere [11]. The experimental group is suspended for 21 days (the period was chosen based on prior studies since it demonstrates changes in organ and body weights after HLU) [10]. The animals are freed from the string after the suspension to allow them to recover and determine acute stress reversal. Our preliminary findings suggest that various biomarkers associated with acute stress were observed in animals during stressful HLU, compared with animals kept under normal conditions, such as vasodilator-stimulated phosphoprotein, myeloperoxidase, high sensitivity C reactive protein, IL-8, plasminogen activator inhibitor-1, intercellular adhesion molecule-1 and monocyte chemoattractant protein-1. The use of the model can also help reveal epigenetic changes using transcriptomic analysis in the brain, gut, liver, muscles, lungs, heart and other tissues. In this regard, the aforementioned animal model is highly advantageous, in that, blood, sweat, cerebrospinal fluid (CSF), tissues, oral and gut microbiome samples can be collected to carryout molecular studies. Blood can be collected by cardiac puncture that allows up to 10 ml of blood from a single 150 g rat. The CSF is collected from cisterna magna of the rats. Briefly, rats are anesthetized and a butterfly needle connected to a syringe is directly punctured into the cisterna magna, and the noncontaminated sample is drawn into the syringe. The tubing is cut with a pair of scissors from the clear part to avoid blood contamination. Then the clear CSF (ca. 100–250 μl per rat) is obtained. For sweat induction and collection, three washes of distilled water are used to clean the footpads of anesthetized rats. The next step is to submerge the footpads in light mineral oil to stop evaporative losses. Next, pilocarpine (10 mg/kg) in water is injected subcutaneously dorsal to the scapula to induce sweating. Sweat is collected using a 20–70 nl constriction pipette, with mineral oil acting as a constant vapor barrier [12]. For epigenetic studies, animals are euthanized and gastrocnemius muscles, brain, heart and other organs are carefully dissected, weighed and subjected to DNA methylation studies. Additionally, the aforementioned model has been used by NASA as a common model to study simulated spaceflight induced weightlessness and cephalad fluid shift in animals is the hindlimb unloading model.

In summary, acute stress can affect our autonomic, endocrine and musculoskeletal performance (reviewed in [13]) as well as the gut microbiome dysbiosis that plays a significant role in regulating the behavior and health of its host [14]. The development of interventional strategies and/or reversal of pathophysiological effects in response to acute stress, requires a complete understanding of the underlying molecular mechanisms at the physiological and oral/gut microbiome level. A holistic approach using whole organisms involving metagenomics, metabolomics, metaproteomics and epigenetics is crucial to comprehend higher order processes as well as motor skills in response to acute stress. In this regard, the use of proposed model for has the potential to identify potential biomarkers involved in acute stress. If the agents identified are novel and the effects can be reversed, the outcomes may ultimately result in the development of new prebiotics, probiotics and postbiotics to improve human health. This will have a huge clinical impact worldwide, in diagnostic, therapeutics and/or risk prediction models. Overall, it is anticipated that the knowledge gained from such research has the potential to identify novel molecules that could be exploited for human health and performance, and it is a priority area of research.

## References

[1] Harari YN. Sapiens: A Brief History of Humankind. Harper, NY, USA (2015).

[2] Lehner AF, Rumbeiha W, Shlosberg A Diagnostic analysis of veterinary dried blood spots for toxic heavy metals exposure. J. Anal. Toxicol. 37(7), 406–422 (2013). 23861340 10.1093/jat/bkt048

[3] Schneider L, Peleja RP, Vogt RC, Da Silveira R. Mercury concentration in the spectacled caiman and black caiman (Alligatoridae) of the Amazon: implications for human health. Arch. Environ. Contam. Toxicol. 63(2), 270–279 (2012). 22580737 10.1007/s00244-012-9768-1

[4] Siddiqui R, Jeyamogan S, Ali SM, Abbas F, Sagathevan KA, Khan NA. Crocodiles and alligators: antiamoebic and antitumor compounds of crocodiles. Exp. Parasitol. 183, 194–200 (2017). 28917711 10.1016/j.exppara.2017.09.008

[5] Khan NA, Soopramanien M, Maciver SK, Anuar TS, Sagathevan K, Siddiqui R. *Crocodylus porosus* gut bacteria: a possible source of novel metabolites. Molecules 26(16), 4999 (2021). 34443585 10.3390/molecules26164999 PMC8398445

[6] Valles-Colomer M, Blanco-Míguez A, Manghi P *et.al*. The person-to-person transmission landscape of the gut and oral microbiomes. Nature. 614(7946), 125–135 (2023). 36653448 10.1038/s41586-022-05620-1 PMC9892008

[7] Dutta S, Sengupta P. Men and mice: relating their ages. Life Sci. 152, 244–248 (2016). 26596563 10.1016/j.lfs.2015.10.025

[8] Hagan C. When are mice considered old. The Jackson Laboratory. 649 (2017). www.jax.org/news-and-insights/jax-blog/2017/november/when-are-mice-considered-old#:~:text=Mice%20ranging%20from%2018%20%2D%2024,all%20biomarkers%20in%20all%20animals

[9] Morey-Holton ER1, Globus RK. Hindlimb unloading rodent model: technical aspects. J. Appl. Physiol. 92(4), 1367–1377 (2002). 11895999 10.1152/japplphysiol.00969.2001

[10] Shama S, Qaisar R, Khan NA, Tauseef I, Siddiqui R. The role of 4-phenylbutyric acid in gut microbial dysbiosis in a mouse model of simulated microgravity. Life (Basel) 12(9), 1301 (2022). 36143337 10.3390/life12091301 PMC9503658

[11] Burkholder T, Foltz C, Karlsson E, Linton CG, Smith JM. Health evaluation of experimental laboratory mice. Curr. Protoc. Mouse Biol. 2(2), 145–165 (2012). 22822473 10.1002/9780470942390.mo110217 PMC3399545

[12] Keller RW, Bailey JL, Wang Y, Klein JD, Sands JM. Urea transporters and sweat response to uremia. Physiol. Rep. 4(11), e12825 (2016). 27273880 10.14814/phy2.12825 PMC4908500

[13] Anderson GS, Di Nota PM, Metz GAS, Andersen JP. The impact of acute stress physiology on skilled motor performance: implications for policing. Front. Psychol. 10, 2501 (2019). 31781001 10.3389/fpsyg.2019.02501 PMC6856650

[14] Heyde KC, Ruder WC. Exploring host–microbiome interactions using an *in silico* model of biomimetic robots and engineered living cells. Sci Rep. 5, 11988 (2015). 26178309 10.1038/srep11988 PMC4648426

